# Label-free electrochemical immunosensor based on enhanced signal amplification between Au@Pd and CoFe_2_O_4_/graphene nanohybrid

**DOI:** 10.1038/srep23391

**Published:** 2016-03-18

**Authors:** Yong Zhang, Jiaojiao Li, Zhiling Wang, Hongmin Ma, Dan Wu, Qianhe Cheng, Qin Wei

**Affiliations:** 1 Key Laboratory of Chemical Sensing & Analysis in Universities of Shandong, School of Chemistry and Chemical Engineering, University of Jinan, Jinan 250022, PR China

## Abstract

The improvement of sensitivity of electrochemical immunosensor can be achieved via two approaches: increasing loading capacities of antibody and enlarging responding electrochemical signals. Based on these, CoFe_2_O_4_/graphene nanohybrid (CoFe_2_O_4_/rGO) as support was firstly used for preparing electrochemical biosensor, and with the addition of Au@Pd nanorods (NRs) as mimic enzyme, a label-free electrochemical immunosensor was prepared. Due to the high electrical conductivity, open porous structure and large loading capacities of CoFe_2_O_4_/rGO, the enhanced signal amplification between Au@Pd NRs and CoFe_2_O_4_/rGO was studied. Fabricated as a novel substrate, the prepared immunosensor had a good analytical performance and exhibited a wide linear range from 0.01 to 18.0 ng·mL^−1^ with a low detection limit of 3.3 pg·mL^−1^ for estradiol, which was succeeded in applying to detect estradiol in the natural water.

Estradiol is a type of environmental estrogens which are chemicals with similar biological activity of estrogens in the body and the environment. Estradiol is a naturally-occurring steroid hormone and widely exists in the natural water. The presence of estradiol in the natural water can disrupt the endocrine, immune, nervous and other systems of humans and cause abnormal sexual development^1^,^2^,^3^. As such, it is a kind of serious environmental toxins to human health^4^. Therefore, the methods of sensitively detecting estradiol have recently attracted the attention of the scientific community and researchers.

So far, various highly sensitive techniques for the detection of estradiol have been extensively developed, such as liquid chromatography (LC), mass spectrometry (MS), gas chromatography (GC), solid-phase extraction-enzyme-linked immunosorbent assay (SPE-ELISA), GC-MS, and LC-MS^5^,^6^,^7^,^8^. Compared with these techniques, electrochemical method has many advantages of ease of operation and low cost^9^,^10^,^11^,^12^. For this reason, several studies have focused on fabricating electrochemical immunosensor for ultrasensitive and rapid detection of estradiol in recent years^13^,^14^. With respect to the strategy of electrochemical immunosensor, electrochemical methods based on enzyme or mimic enzyme to realize the signal amplification of antigen and antibody reactions have been mostly studied. The use of robust and stable nanoparticles (NPs) as mimic enzymes has received widespread attention, especially noble metal NPs^15^,^16^,^17^. Especially, Au NPs have been regarded as a type of versatile template for the immobilization of biomolecules and also play important roles in numerous fields of biomedical applications, due to their advantages, such as easy preparation, high homogeneity, and biocompatibility with antibody or antigen^18^,^19^,^20^. In addition, Pd and Ag NPs with superior electrochemical properties can facilitate the electron transfer from the redox center of protein to the electrode surface. Therefore, the bimetallic NPs, such as Au/Ag and Au/Pd also attract widespread attention especially in the field of immunosensors, and they often show much better electrochemical performance than their monometallic counterparts^21^.

More recently, we fabricated a sandwich-type electrochemical immunosensor for detection of estradiol^22^. Compared with sandwich-type, label-free electrochemical immunosensor has attracted great research interests due to its simple preparation, low cost and exemption of use of secondary antibody. The functions of established label-free electrochemical immunosensor are accomplished by a main mechanism that the steric hindrance produced from the formation of immunocomplexes can prevent electrons from reaching the surface of the electrode and increase resistance, so as to realize the quantitative and qualitative detection of the antigen^23^. Therefore, to get good analytical performance of label-free electrochemical immunosensor, the capacities for analyte loading and abilities need to be improved for signal amplification.

Nanohybrids of graphene sheets loaded with spinel oxides, such as Co_3_O_4_/graphene^24^, MnCo_2_O_4_/graphene^25^, NiCo_2_O_4_/graphene^26^, have been reported to be promising bi-functional electrocatalysts for the oxygen reduction reaction (ORR). Due to the high electrical conductivity, chemical stability, large surface area and open porous structure of these nanohybrids^27^,^28^, they might be used as the substrate for fabricating label-free electrochemical immunosensor.

In this work, CoFe_2_O_4_/graphene nanohybrid (CoFe_2_O_4_/rGO) was first synthesized and modified onto the electrode to enlarge the specific surface area and electrical conductivity. As shown in Fig. 1, after Au@Pd core@shell nanorods (Au@Pd NRs) as mimic enzymes are in successive fabricated onto the film of CoFe_2_O_4_/rGO, the substrate of the label-free immunosensor is prepared. Due to the excellent electron transport capability and strong adsorption capacity of CoFe_2_O_4_/rGO, the remarkable improvement of the immobilizing amount of antibody on the electrode surface and great enhancement of the electrical signal of the substrate of CoFe_2_O_4_/rGO and Au@Pd NRs are achieved, and then a label-free electrochemical immunosensor for detecting estradiol is prepared.

## Discussion

### Characterization of Au NRs; Au@Pd NRs and CoFe_2_O_4_/rGO

The morphology of Au NRs and Au@Pd NRs were characterized through TEM and SEM. As illustrated in Fig. 2A,B, Au NRs with a uniform aspect ratio of approximately 3.6 can be seen. After coating Pd NPs, bone-like Au@Pd NRs with single-crystal structure are formed for Pd preferentially adopting epitaxial growth on Au surface, as presented in Fig. 2C,D.

To better know the preparation CoFe_2_O_4_/rGO nanohybrid, the as-prepared graphite oxide (GO) and CoFe_2_O_4_/rGO were characterized through TEM, SEM and XRD. From the TEM and SEM images of GO in Fig. 3A,B, they are confirmed that the shape of GO is multilayer and thin wrinkled paper-like structure. After *in situ* growth of CoFe_2_O_4_ with reduction of GO, the average crystallite size of the CoFe_2_O_4_ particles, which are observed in Fig. 3C, is estimated to be 4.9 nm. Besides, the XRD profiles of CoFe_2_O_4_/rGO can be well indexed as cubic spinel phase (PDF#22-1086) except the broad peak at around 23° corresponding to (002) peak of carbon in GO (Fig. 3D).

### Characterization of fabricating the immunosensor and the enhanced signal amplification between Au@Pd NRs and CoFe_2_O_4_/rGO

The assembly process of the immunosensor was monitored by electrochemical impedance spectroscopy (EIS), which had been proven to be one of most powerful tools for probing the features of surface-modified electrodes^29^. As shown in Fig. 4A, the bare glassy carbon electrode (GCE) exhibits an almost straight line (curve a), which is characteristic of a diffusional limiting step of the electrochemical process. As expected, the EIS curves of the CoFe_2_O_4_/rGO and Au@Pd NRs successively modified electrodes (curve b and c) are similar to that of the bare GCE. The reason is that the CoFe_2_O_4_/rGO and Au@Pd NRs immobilized on the electrode are similar to a conducting film, which makes it easier for the electron transfer. After the modified electrode is incubated in estradiol antibody (anti-EST, curve d) and bovine serum albumin (BSA, curve e), successively, which can hinder the electron-transfer of the electrochemical probe, the value of resistance is getting increased. This indicates the successful immobilization of anti-EST. Furthermore, the resistance increase again (curve f), after the capture of estradiol, indicating estradiol/anti-EST immunocomplex is well formed. Thus, we concluded that this strategy could provide a sensitive electric interface for further sensing.

As we stated previously, the improvement of sensitivity of electrochemical immunosensor could be achieved via two approaches: increasing loading capacities of antibody and enlarging responding electrochemical signals. As shown in Fig. 4A, the sensing mechanism of this strategy can be divided into two parts. Because CoFe_2_O_4_/rGO (curve b) and Au@Pd NRs (curve c) are all electrical active materials, no obvious difference between them can be found in the electrolyte without H_2_O_2_. However, with the addition of several types of biomolecules step by step, distinct decrease of signals can be observed in the electrolyte without H_2_O_2_. Based on this phenomenon, the first part is the increase of electrochemical signals responded with the electrical catalysis to H_2_O_2_, which can be enhanced by different electrical catalyst materials (Fig. 4B). The second part is the decrease of electrochemical signals responded with the increase of electrical resistance emerged by immunocomplex of the biomolecules, which can be enhanced by electrode modifying substrate of large loading capacities (inset of Fig. 4A).

In order to investigate the enhancing the loading capacity effect of CoFe_2_O_4_/rGO in this strategy, the capacities of loading biomolecules by EIS spectrogram analysis between the electrodes modified with and without CoFe_2_O_4_/rGO were compared. As shown in the inset of Fig. 4A, after the immobilization of anti-EST on the electrode with (curve d) or without (curve g) CoFe_2_O_4_/rGO modifying, it is delightful to find obvious decrease of the resistance value, which demonstrates the key effect of CoFe_2_O_4_/rGO for enhancing the analyte loading capacities. Therefore, CoFe_2_O_4_/rGO plays a major role in the strategy for enhancing the loading capacity of the biomolecules of the immunosensor.

As well known, catalytic effect of Pd catalysts for improved H_2_O_2_-electroreduction can be improved through synthesizing Pd-based bimetallic NPs and well dispersing upon various electron-conductive nanomaterials. However, to find new substrates and bimetallic catalysts for H_2_O_2_-electroreduction is still challenge in nanomaterial field. Furthermore, electrochemical analysts always pay more attention to study the enhanced signal amplification between the substrate and catalyst and to fabricate novel electrochemical immunosensing strategies. Herein, for better investigating the enhanced signal amplification between Au@Pd NRs and CoFe_2_O_4_/rGO, three different types of modified electrodes were studied in electrocatalysis to H_2_O_2_, including Au@Pd NRs, CoFe_2_O_4_/rGO, both of Au@Pd NRs and CoFe_2_O_4_/rGO. As shown in Fig. 4B, the amperometric signal of the electrode modified with both of Au@Pd NRs and CoFe_2_O_4_/rGO (curve d) is much greater than those with Au@Pd NRs and CoFe_2_O_4_/rGO respectively (curve b and curve c), which provides a powerful evidence of the designed signal amplification strategy. The amplified response may originate from the employment of the CoFe_2_O_4_/rGO with large surface area and open porous structure, which not only can enhance the electrical conductivity and electrical catalysis for ORR^14^, but also can load more Au@Pd NRs with large surface area and excellent catalytic performance^30^. This effect also can be investigated via cyclic voltammetry (CV) responses for H_2_O_2_ reduction at different electrodes (curve a, e and f in Fig. S1). As expected, after the biomolecules are modified onto the electrode, the amperometric signals (curve b–d in Fig. S1) corresponding to the electrical catalysis to H_2_O_2_ will be decreased, due to the increase of electrical resistance mentioned above.

### Optimization of experimental conditions

To get the best performance of the immunosensor, experimental conditions include the pH value and the concentration selection of Au@Pd NRs and CoFe_2_O_4_/rGO were optimized.

As shown in Fig. 5A, with the increase of pH of phosphate buffered saline (PBS) from 3.0 to 10, the current response of the electrode to 10 ng·mL^−1^ estradiol is increased firstly and reached the maximum at 7.4 but then decreased. So the pH value of 7.4 was selected as optimal condition for the detection. As seen in Fig. 5B, the current response is mainly influenced by the concentration of CoFe_2_O_4_/rGO. If CoFe_2_O_4_/rGO is not used for the fabrication of the immunosensor, there is very small electrochemical signal, which was only resulting from Au@Pd NRs. With the concentration of CoFe_2_O_4_/rGO increased, the current response is increased and get highest at the 10 mg·mL^−1^ and with no obvious change until 15 mg·mL^−1^. As stated previously, the main reason is the enhanced signal amplification between Au@Pd NRs and CoFe_2_O_4_/rGO. Considering the cost, 10 mg·mL^−1^ of the concentration of CoFe_2_O_4_/rGO was chosen.

Under the optimum conditions, the fabricated label-free immunosensor was used for detecting different concentrations of estradiol through the amperometric measurement with a detection potential at −0.3 V. After the background current was stabilized, 1.0 mmol·L^−1^ H_2_O_2_ was added into the buffer and the current change was recorded. Figure 6A shows that the relationship between current change and the concentration of estradiol, and the current is decreased linearly within the estradiol ranging from 0.01 to 18.0 ng·mL^−1^. The equation of the calibration curve is Y = 10.75 + 5.886 X, R = 0.998 and a low detection limit (3.3 pg·mL^−1^) of the immunosensor is obtained. Compared with the previous reports on electrochemical immunosensors for estradiol detection, our work obtains much better performance as listed in Table S1^31^,^32^,^33^. These results are attributed to two factors, the enhancement of the capacities for analyte loading by CoFe_2_O_4_/rGO and the amplification of the electrochemical signal due to the enhanced signal amplification between Au@Pd NRs and CoFe_2_O_4_/rGO, both of which increased the sensitivity and improve electrical signal.

### Reproducibility, stability and selectivity of the immunosensor

To study the reproducibility of the immunosensor, a series of five electrodes were prepared for the detection of estradiol. The Relative standard deviation (RSD) of the measurements for five immunosensors is less than 3.5%. The results suggest that the precision of the proposed immunosensor is satisfying.

The stability of the immunosensor was also measured. The electrodes were stored at 4 °C for further usage. A week later, the current response of immunosensor had no apparent change. The current response of the immunosensor decreased 5.0% after half a month. The good stability is ascribed to two reasons: Au@Pd NRs and CoFe_2_O_4_/rGO have good stability and the antibody can firmly bind with the Au@Pd NRs with perfect biocompatibility through covalent binding of noble metal NPs^34^.

To evaluate the selectivity and specificity of the system, possible interferents including pyrogallolred (column b), naphthol (column c), fluorone black (column d), 1-(2-Pyridylazo)-2-naphthol (column e) and diethylstilbestrol (column f) were investigated and the stradiol solutions (10 ng·mL^−1^) containing 1 μg·mL^−1^ of interfering substances were measured by the immunosensor. As shown in Fig. 6B, the current variation exhibits no significant influence (3%) caused by addition of the interfering substances compared with pure estradiol solution of 10 ng·mL^−1^ (defined as “blank”, column a), which indicats that the proposed label-free method has a good specificity.

### Real sample analysis

The analytical reliability and application feasibility of the prepared immunosensor were demonstrated by standard addition methods. The recoveries of different concentrations (1.0, 5.0 and 10.0 ng·mL^−1^) of estradiol were detected in the river water, which was sampled from University of Jinan. The water was extracted by 0.2 mm micro pore filter. The extract was then diluted with PBS (pH = 7.4). The recovery standard addition is 97.8–99.1% and the RSD is in the range of 3.54–6.21%. The results are listed in Table S2 and indicate that the proposed method exhibits great potential as a reliable technique for the determination of estradiol in environmental water samples.

## Conclusions

In this work, CoFe_2_O_4_/rGO is firstly used for preparing electrochemical biosensor, and with the addition of Au@Pd NRs, a label-free electrochemical immunosensor has been prepared and applied for detection of estradiol, which gets good analytical performances, such as sensitivity, reproducibility, stability *et al.* These good results are owing to two reasons. First, the high electrical conductivity, chemical stability, large surface area and open porous structure of CoFe_2_O_4_/rGO can increase the capacity of loading Au@Pd NRs and biomolecules and the rate of electron-transfer. Second, the current response to H_2_O_2_ due to the enhanced signal amplification between Au@Pd NRs and CoFe_2_O_4_/rGO can be greatly improved. Furthermore, the prepared immunosensor has been successful in applying to detect estradiol in the river water.

## Methods

### Chemicals and reagents

All chemicals were used as-received without further processing. HAuCl_4_·3H_2_O, AgNO_3_ and K_2_PdCl_4_ were obtained from Shanghai Reagent Co. (Shanghai, China) and used as received. Cetyltrimethylammonium bromide (CTAB), glutaraldehyde (GA), ascorbic acid (AA), Fe(NO_3_)_3_·9H_2_O, Co(NO_3_)_2_·6H_2_O, NH_4_OH and NaOH were provided by Sinopharm Chemical Reagent Co. (China). BSA (96–99%) was purchased from Sigma (USA) and used as received. Estradiol antigen and anti-EST were purchased from Wanger Biotechnology Co., Ltd. (Beijing, China). All other reagents were of analytical grade. PBS (0.1 mol·L^−1^ containing 0.1 mol·L^−1^ NaCl) was used as electrolyte for all electrochemistry measurements. Ultra-pure water was prepared by a Millipore Milli-Q system and used throughout.

### Apparatus

All electrochemical measurements were performed on CHI760D electrochemical workstation (Shanghai Chenhua Instrument Co. Ltd., China). EIS was obtained from the impedance measurement unit (IM6e, ZAHNER elektrik, Germany). All electrochemical experiments were carried out in a conventional three-electrode cell with a modified GCE (diameter 4 mm) as the working electrode, a Pt wire electrode as the counter electrode and an Ag/AgCl electrode as the reference electrode. The UV-Vis absorption spectra of water colloid were recorded on Shimadzu UV3600 UV-Vis-NIR spectrophotometer (Lumerical Solutions, Inc.). XRD patterns of the prepared samples were acquired with a Rigaku D/MAX 2200 X-ray diffractometer (Tokyo, Japan) (Bragg equation 2*d* sin *θ* = *nλ*, *n* = 1, *λ* = 0.154 nm). Transmission electron microscopy (TEM, JEOL JEM 1200EX working at 100 kV) and high-resolution TEM (HRTEM, FEI Tecnai G2 F20 S-Twin working at 200 kV) were utilized to characterize morphology and interfacial lattice details. Scanning electron microscope (SEM) images were obtained using a field emission SEM (Zeiss, Germany).

### Preparation of Au NRs

Au NRs were synthesized via a typical seed mediated growth method with some modifications^35^,^36^. Briefly, Au-seeds aqueous solution was firstly prepared by adding 0.6 mL of ice-cold 10 mmol·L^−1^ NaBH_4_ to 10 mL of solution containing 0.10 mol·L^−1^ CTAB and 0.25 mmol·L^−1^ HAuCl_4_. After vigorous stirring for 30 s, the resulted brownish yellow solution was undisturbed at 25 °C for 30 min. Then, the growth solution was prepared via successively adding 0.35 g KBr, 1.2mL of 4 mmol·L^−1^ AgNO_3_, 25 mL of 1 mmol·L^−1^ HAuCl_4_ and 0.45 mL of 64 mmol·L^−1^ AA to 0.10 mol·L^−1^ CTAB aqueous with stirring in the whole procedure. At last, 0.08 mL Au-Seeds was added to the growth solution and undisturbed at 25 °C for 12 h, with the color changing from colorless to dark blue. After the solution was centrifuged (6000 rpm for 15 min) twice with washing by deionized water, the resulted precipitates were re-dispersed in 5 mL deionized water to get Au NRs colloidal sol.

### Preparation of Au@Pd NRs

Au@Pd NRs were synthesized simply via the following steps: 300 μL of 0.1 mol·L^−1^ AA, 50 μL of 10 mol·L^−1^ K_2_PdCl_4_, and 300 μL of 0.1 mol·L^−1^ NaOH were successively added to 5 mL Au NRs colloidal sol under the condition of stirring at 25 °C. After 30 min, the Au@Pd NRs solution was centrifuged (6000 rpm for 15 min) followed by consecutive washing three times with deionized water and the resulted precipitates were re-dispersed in 5 mL deionized water to get Au@Pd NRs colloidal sol.

### Preparation of CoFe_2_O_4_/rGO

CoFe_2_O_4_/rGO was prepared using the previously reported method^27^. Firstly, graphene oxide (GO) powders were synthesized from graphite by a modified Hummers’ method as described elsewhere^37^,^38^. After centrifugation followed by lyophilization, 10 mg GO was dispersed in 30 mL anhydrous ethanol with sonication for 1 h to get GO ethanol suspension. Secondly, 1 ml of 0.2 mol·L^−1^ Fe(NO_3_)_3_·9H_2_O and 0.5 ml of 0.2 mol·L^−1^ Co(NO_3_)_2_·6H_2_O aqueous solution was added to the above GO ethanol suspension, followed by the addition of 2.50 ml of NH_4_OH at room temperature. The mixture was stirred at 80 °C for 12 h and then transferred to a 50 mL autoclave, sealed and heated at 180 °C for 5 h. Finally, after centrifugation and wash with ethanol and deionized water, the final product of CoFe_2_O_4_/rGO was obtained after lyophilization.

### Fabrication of the immunosensor

**Figure 1:**
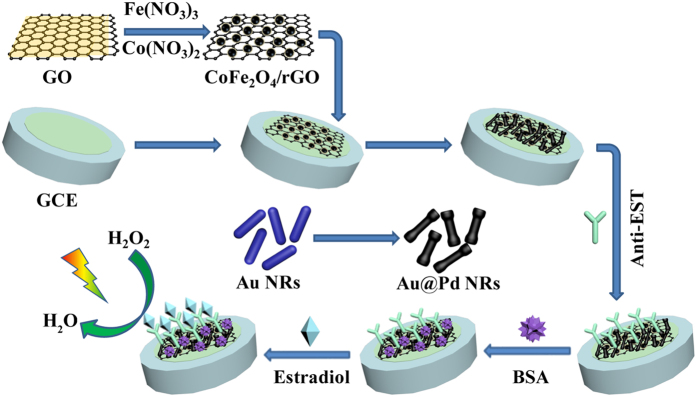
The fabrication procedure of the label-free electrochemical immunosensor.

**Figure 2:**
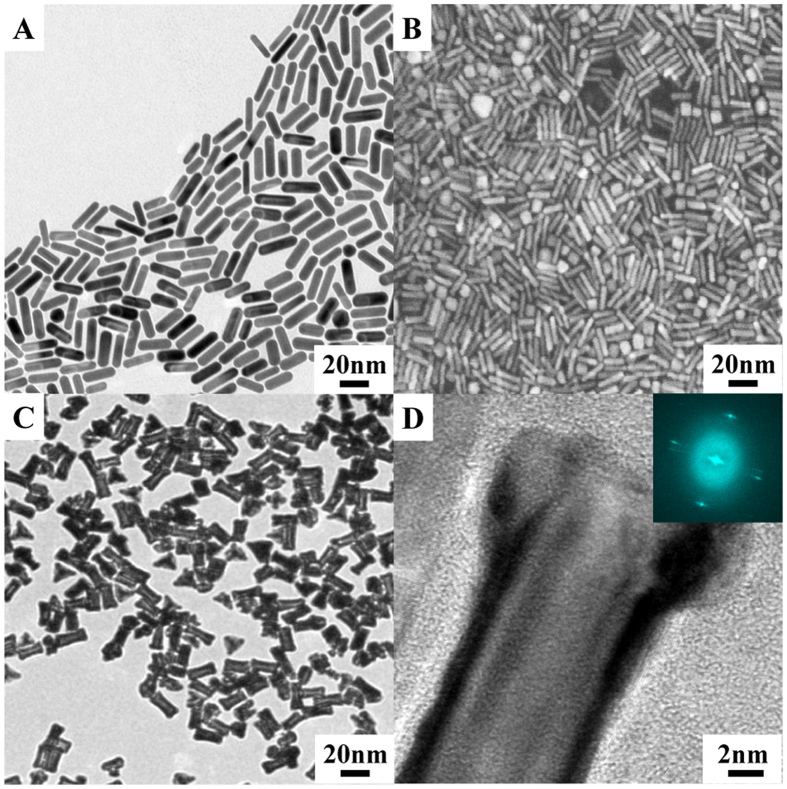
(**A**) TEM image and (**B**) SEM image of Au NRs. (**C**) TEM image of Au@Pd NRs, local zoom (inset). (**D**) HRTEM image of Au@Pd NRs, the FFT pattern (inset).

**Figure 3:**
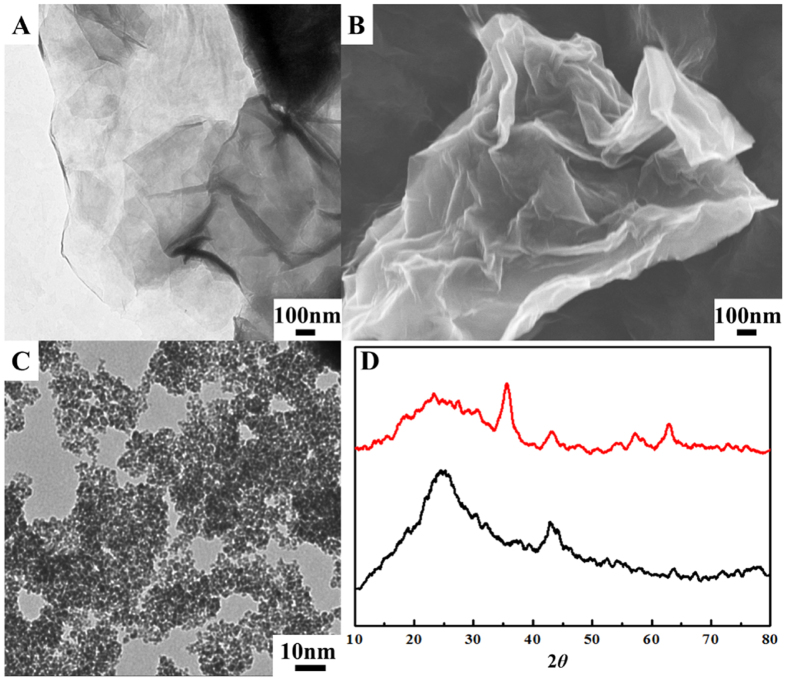
(**A**) TEM image and (**B**) SEM image of GO. (**C**) TEM image of CoFe_2_O_4_/rGO. (**D**) XRD pattern of GO (black) and CoFe_2_O_4_/rGO (red).

**Figure 4:**
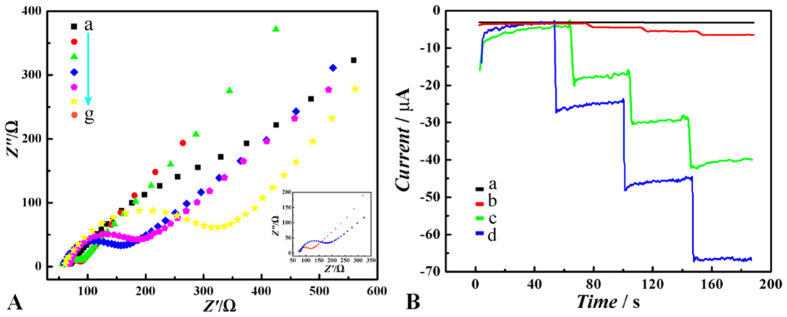
(**A**) Nyquist plots of the EIS for each immobilized step of (a) bare GCE, (b) CoFe_2_O_4_/rGO/GCE, (c) Au@Pd/CoFe_2_O_4_/rGO/GCE, (d) anti-EST/Au@Pd/CoFe_2_O_4_/rGO/GCE, (e) BSA/anti-EST/Au@Pd/CoFe_2_O_4_/rGO/GCE, (f) estradiol/BSA/anti-EST/Au@Pd/CoFe_2_O_4_/rGO/GCE in 1 mmol·mL^−1^ Fe(CN)_6_^3-/4-^ solution. (**B**) Amperometric response at −0.3 V vs. SCE for the GCE with different materials modified toward successive addition of 100 μL 1.0 mmol·L^−1^ H_2_O_2_ into pH7.4 PBS buffer solution (0.1 mol•L^−1^ containing 0.1 mol•L^−1^ NaCl): (a) bare GCE, (b) CoFe_2_O_4_/rGO/GCE, (c) Au@Pd/GCE, (d) Au@Pd/CoFe_2_O_4_/rGO/GCE.

**Figure 5:**
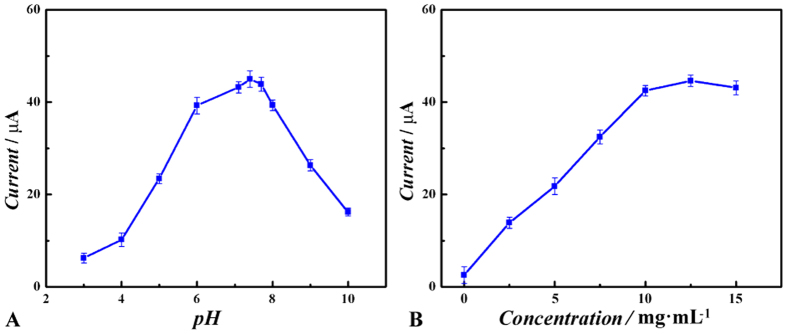
Optimization of the experimental parameters: effects of (**A**) pH value and (**B**) Au@Pd NRs ratio on the response of the label-free electrochemical immunosensor, amperometric response at−0.3 V vs. SCE with the addition of 100 μL 1.0 mmol·L^−1^ H_2_O_2_.

**Figure 6:**
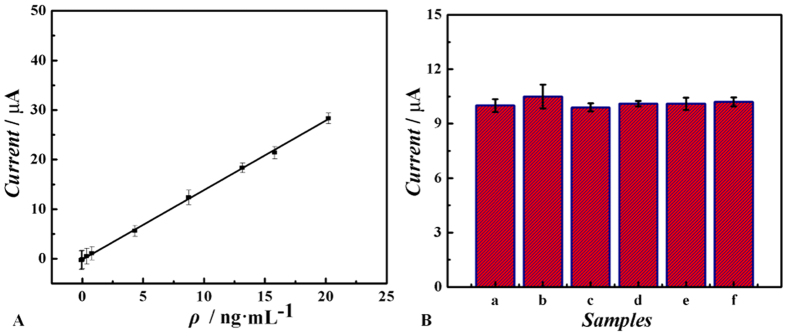
(**A**) Calibration curve of the immunosensor for different concentrations of estradiol. (**B**) A comparison of amperometric responses of 10 ng·mL^−1^ estradiol with those of 100 ng·mL^−1^ different interfering species: (a) blank, (b) pyrogallolred, (c) naphthol, (d) fluorone black, (e) 1-(2-Pyridylazo)-2-naphthol and (f) diethylstilbestrol, amperometric response at−0.3 V vs. SCE with the addition of 100 μL 1.0 mmol·L^−1^ H_2_O_2_.

Figure 1 illustrates the procedure of the fabrication of the immunosensor. Firstly, the bare GCE is polished with 1.0, 0.3 and 0.05 μm alumina powder and washed with ultrapure water for a few seconds. Then, 6 μL of the CoFe_2_O_4_/rGO and 6 μL of the Au@Pd NRs in succession are dispersed onto the electrode surface via physical adsorption and dried in the room temperature. Afterwards, 6 μL of anti-EST (10 μg·mL^−1^) is adsorbed on the electrode surface and incubated at 4 °C for 1 h. Subsequently, the electrode is washed and 3 μL of BSA (1%) is dispersed onto the electrode for another 30 min to block nonspecific binding sites. Finally, different concentrations of estradiol solutions are dropped onto the electrodes and incubated for 1 h. After washing, the electrodes are ready to be measured.

### Measurement procedure

A conventional three-electrode system was used for all electrochemical measurements: GCE as the working electrode, a saturated calomel electrode (SCE) as the reference electrode, and a platinum wire electrode as the counter electrode. The pH 7.4 PBS buffer was used for all the electrochemical measurements. CV responses were recorded in 10 mL of PBS at 100 mV·s^−1^. For amperometric measurement, a detection potential of −0.3 V was selected and after the background current was stabilized, 1.0 mmol·L^−1^ H_2_O_2_ was added into 10 mL of the buffer and the current change was recorded.

## Additional Information

**How to cite this article**: Zhang, Y. *et al.* Label-free electrochemical immunosensor based on enhanced signal amplification between Au@Pd and CoFe_2_O_4_/graphene nanohybrid. *Sci. Rep.* **6**, 23391; doi: 10.1038/srep23391 (2016).

## Supplementary Material

Supplementary Information
