# Supplementary material for: Label-free electrochemical immunosensor based on enhanced signal amplification between Au@Pd and CoFe2O4/graphene nanohybrid
Source: Sci Rep. 2016 Mar 18;6:23391. doi: 10.1038/srep23391 (PMC4796816; doi:10.1038/srep23391)
Supplement: Supplementary Information [file srep23391-s1.doc]

**Supporting Information**

*of*

**Label-free electrochemical immunosensor based on enhanced signal amplification between Au@Pd and CoFe_2_O_4_/graphene nanohybrid**

Yong Zhang, Jiaojiao Li, Zhiling Wang, Hongmin Ma, Dan Wu, Qianhe Cheng and Qin Wei^*^

Key Laboratory of Chemical Sensing & Analysis in Universities of Shandong, School of Chemistry and Chemical Engineering, University of Jinan, Jinan 250022, PR China

Yong Zhang (E-mail: yongzhang7805@126.com)

Jiaojiao Li (E-mail: jndxlijj@163.com)

Zhiling Wang (E-mail: 470887540@qq.com)

Hongmin Ma (E-mail: mahongmin2002@126.com)

Dan Wu (E-mail: wudan791108@163.com)

Qianhe Cheng (E-mail: 746581632@qq.com)

Qin Wei^*^ (E-mail: sdjndxwq@163.com)

^*^Corresponding author. Tel: + 86 531 82767872; fax: + 86 531 82767367.

E-mail address: sdjndxwq@163.com (Qin Wei).

**
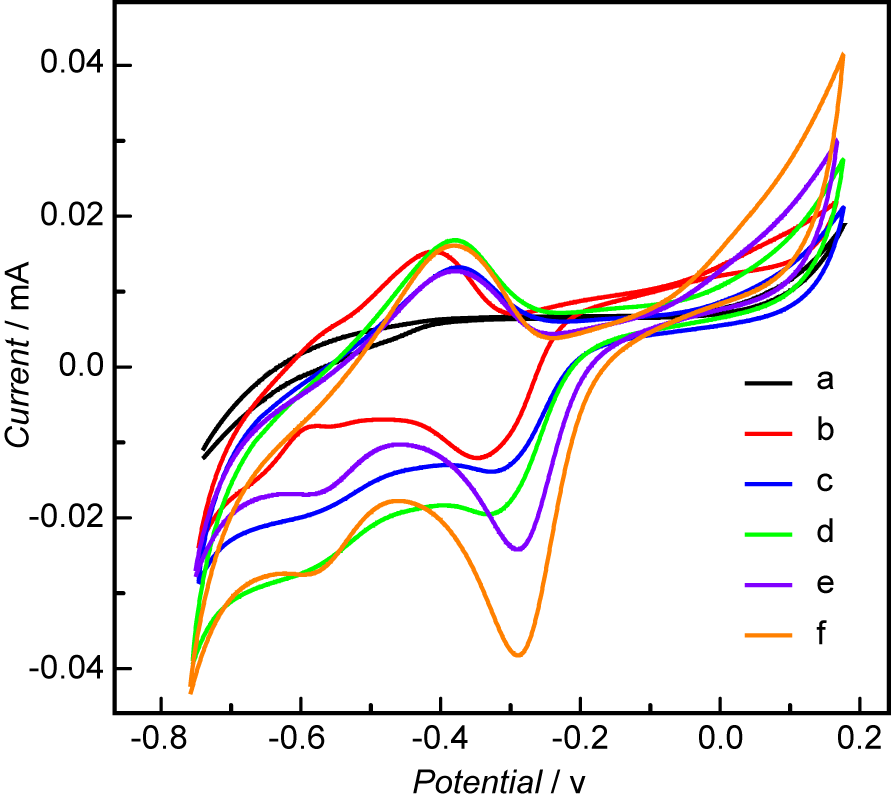
**

Figure S1. CV response curves for different electrodes of (a) bare GCE, (b) estradiol/BSA/anti-EST/Au@Pd/CoFe_2_O_4_/rGO/GCE, (c) BSA/anti-EST/Au@- Pd/CoFe_2_O_4_/rGO/GCE, (d) anti-EST/Au@Pd/CoFe_2_O_4_/rGO/GCE, (e) Au@Pd/GCE, (f) Au@Pd/CoFe_2_O_4_/rGO/GCE with the addition of 100 µL 1.0 mmol·L^-1^ H_2_O_2_ into PBS (pH 7.4) solution, scan rate: 100 mV·s^-1^.

**Table S1. Comparison between the present work and other reported techniques for the determination of estradiol.**

| The material of sensors | Method | linear range (ng·mL^-1^) | detection limit (ng·mL^-1^) | References |
| --- | --- | --- | --- | --- |
| Nickel hexacyanoferrate NPs | DPV | 272~1.36×10^5^ | 217 | 1 |
| 6-(O-carboxy-methyl)oxime-BSA | Fluorescence | 5.4×10^5^~2.2×10^6^ | 5.4×10^5^ | 2 |
| CdSe/TiO_2_ | PEC | 13.6~5.44×10^3^ | 0.8 | 3 |
| Ru(bpy)_3_^2+^ | ECL | 2.7×10^3^~2.7×10^6^ | 299.2 | 4 |
| Cu_2_S | SWV | 0.025~7.5 | 7.5×10^-3^ | 5 |
| This work | I-t | 0.01~18.0 | 0.0033 | - |

**Table S2. The results of the estradiol determination in river water samples**

| Content of estradiol in the sample (ng·mL^-1^) | The addition content  (ng·mL^-1^) | The detection content  (ng·mL^-1^) | RSD  (%) | Recovery  (%) |
| --- | --- | --- | --- | --- |
| 0.5 | 1.0 | 1.3, 1.6, 1.6, 1.4, 1.5 | 0.8 | 99.9 |
|  | 5.0 | 5.9, 5.6, 5.7, 5.3, 5.5 | 4.6 | 101.6 |
|  | 10.0 | 10.5, 10.4, 10.1, 10.2, 10.7 | 2.3 | 98.8 |

**References**

1. Fan, L., Zhao, G., Shi, H., & Liu, M. A simple and label-free aptasensor based on nickel hexacyanoferrate nanoparticles as signal probe for highly sensitive detection of 17*β*-estradiol, *Biosens. Bioelectron.* **68**, 303-309 (2015).

2. Yildirim, N. et al. Aptamer-Based Optical Biosensor For Rapid and Sensitive Detection of 17β-Estradiol In Water Samples, *Environ. Sci. Technol.* **46**, 3288-3294 (2012).

3.  Fan, L. et al. A Femtomolar Level and Highly Selective 17*β*-estradiol Photoelectrochemical Aptasensor Applied in Environmental Water Samples Analysis, *Environ. Sci. Technol.* **48**, 5754-5761 (2014).

4.  Zhang, J. et al. Label-Free and Sensitive Electrochemiluminescence Aptasensor for the Determination of 17*β*-Estradiol Based On a Competitive Assay with cDNA Amplification. *Anal. Methods* **6**, 6796-6801 (2014).

5.  Zhang, S. et al. An Ultrasensitive Electrochemical Immunosensor for Determination of Estradiol Using Coralloid Cu_2_S Nanostructures as Labels. *RSC Adv.* *5*, 6512-6517 (2015).
